# Neck pain burden in China and G20 countries: an analysis of 1990–2021 with a 30-year forecast for China

**DOI:** 10.3389/fpubh.2025.1541353

**Published:** 2025-07-24

**Authors:** Huale Li, Zhichun Chang, Ting Qin, Yanfang Li, Mingren Hu, Xinjing Yang, Jun Li, Yufeng Xie

**Affiliations:** ^1^Shenzhen Hospital (Futian) of Guangzhou University of Chinese Medicine, Shenzhen, China; ^2^The Sixth Clinical Medical College, Guangzhou University of Chinese Medicine, Shenzhen, China; ^3^State Key Laboratory of Quality Research in Chinese Medicines, Faculty of Chinese Medicine, Macau University of Science and Technology, Cotai, Macao SAR, China

**Keywords:** neck pain, incidence, prevalence, disability-adjusted life years, joinpoint regression analysis

## Abstract

**Background:**

Neck pain (NP) represents a significant global public health challenge and is the fourth leading cause of disability in China and among G20 nations. Given the accelerating trends of population ageing and shifts in contemporary lifestyles, the burden of NP is likely to increase, necessitating urgent, comprehensive analysis and the formulation of effective interventions.

**Objectives and methods:**

This study utilizes the 2021 Global Burden of Disease (GBD) database to extract data concerning the incidence, prevalence, DALY rate, ASIR, ASPR, ASDR, and other relevant metrics of noncommunicable diseases in China and G20 countries from 1990 to 2021. The analysis is conducted via the R programming language, with joinpoint regression employed to calculate the APC and AAPC. Additionally, an ARIMA model is utilized to forecast the incidence rate in China over the next 30 years.

**Results:**

In 2021, China reported 10.29 million (95% UI: 8.06–13.03 million) incident cases and 48.37 million (37.66–60.06 million) prevalent cases of NP. The ASIR, ASPR, and ASDR were estimated at 567 (448–699) per 100,000 population, 2,549 (2,007–3,141) per 100,000 population, and 254 (166–357) per 100,000 population, respectively. Among G20 countries, NP affected 28.71 million (22.50–35.54 million) incident cases and 137.66 million (109.04–169.31 million) prevalent cases, with corresponding ASIR, ASPR, and ASDR values of 507 (398–617) per 100,000 population, 2,362 (1,856–2,892) per 100,000 population, and 234 (156–334) per 100,000 population, respectively. The analysis revealed no statistically significant changes in the AAPCs of the ASIR and ASPR for either China or the G20 countries from 19,902,021. Notably, women consistently presented higher ASIR and ASPR values than men did across all the studied populations. Projection models indicate that by 2051, NP incidence rates will remain stable for China and men, whereas women’s rates are anticipated to significantly decline.

**Conclusion:**

The global community must prioritize the burden of NP and promote precision diagnosis and prevention. Policies should balance technological innovation with social support, strengthen legislative protection for high-risk occupations and harsh climates, implement personalized interventions, and reduce the burden at its root.

## 1 Introduction

Neck pain (NP), whether caused by traumatic or nontraumatic factors, is a prevalent and disabling condition leading to considerable self-reports of pain, disability, and ongoing personal and health care burdens worldwide (1). Peripheral neuropathic pain in the neck is defined as mechanical or chemical irritation primarily from a peripheral nervous system injury or disease, usually involving the nerve roots. The most common examples are radicular symptoms and spinal stenosis due to a herniated or bony disc, whereas central neuropathic pain is a painful condition that may be caused by spinal cord pathology (2). In 2016, low back and NP accounted for the highest health care expenditures of 154 conditions in the U.S., estimated at $134.5 billion (3). In 2012, NP caused 25.5 million people in the U.S. to be absent from work, with an average of 11.4 days of absence per person (4). NP causes considerable personal and socioeconomic burdens, and it is one of the top five chronic pains in terms of prevalence and years of disability. Although most acute episodes resolve spontaneously, more than one-third of patients continue to experience mild symptoms or recurrences for more than 1 year, with genetic and psychosocial factors being persistent risk factors (5). According to the GBD 2021 (6), NP is one of the most common musculoskeletal disorders. In the GBD 2021 study, we found that, among musculoskeletal diseases, the burden of disease for NP in terms of incidence rate, prevalence rate, and DALY rate is relatively high worldwide, whereas it is more pronounced in China and G20 countries. In addition, the impact on the daily life of patients with NP increases with the age of the patients (7). In consideration of the currently accelerating global ageing population, we expect the probability of a reduced quality of life and burden of disability on patients caused by NP to be much greater. However, NP differs from fatal disease because it is nonfatal and primarily affects quality of life through symptoms such as pain and discomfort. Thus, the damage caused by NP, compared with fatal disease, is perceived to be less important (4).

In today’s world, with the rapid improvement in medical care, the life expectancy of human beings will be longer than ever before. China is one of the world’s most populous countries; therefore, it is also one of the countries with the largest and fastest ageing populations in the world. According to key indicators from China’s seventh national census in 2021, China’s population has grown to more than 1.4 billion, and owing to the changing demographic structure, the proportion of older adult people will be greater than ever (8); this means that some age-related diseases, such as NP, are placing an increasing disease burden on the health system. In China, neck pain is one of the most common diseases among people, especially middle-aged and older adults (9). Nearly half of older adults in China suffer from chronic pain (10). In 1990, NP was the 21st leading cause of DALY in China. A cross-sectional study analysing the prevalence and risk factors for musculoskeletal disorders associated with workers in the electronics manufacturing industry in China revealed that the prevalence of musculoskeletal pain is high in the Chinese electronics manufacturing industry and that the impact on work productivity remains significant (11). Currently, if we want to realize the aspiration of “Healthy China 2030,” we must face the difficulties and setbacks encountered during the building a beautiful blueprint for a healthy China and do our best to overcome them (8). Therefore, it is urgent to study the long-term trend of the NP disease burden in recent decades to determine the gap between the current situation and the goal of “Healthy China 2030.” Some previous studies have reported global prevalence rates, years lived with disability (YLD), and projected prevalence rates in China by 2050 from 1990 to 2020 using data from GBD 2021 but not the prevalence rate, DALY rate, and projected incidence rate in China by 2051 for NP (6). In addition, this study did not apply joinpoint regression analysis to analyse the temporal trend of NP for each period or the overall time trend, which is a method that can help policy-makers formulate policies and set future goals.

## 2 Materials and methods

### 2.1 Definitions

GBD 2021 defines NP (ICD-10 code: M54.2, ICD-9 code: 723.1) as pain in the region of the cervical spine (with or without pain in the upper extremities) that lasts for at least 1 day (12) and categorizes types of NP into neuropathic and mechanical. Patients with neuropathic pain often describe their symptoms as shooting, electric, stabbing, and/or burning pain, whereas mechanical pain is more often described as throbbing or aching (2). NP is, of course, usually attributed to injury or disease of the nervous system (neuropathic pain), but it can also stem from injurious (involved) or injurious underlying sources (13). Unique risk factors for NP include traumatic injuries (e.g., traumatic brain injuries and whiplash injuries) and certain sports injuries (e.g., wrestling, hockey, soccer) (14). The age-standardized incidence rate (ASIR) expresses the number of age-standardized new cases of a disease per 100,000 population, which eliminates the effect of differences in age distribution across regions or periods by adjusting the raw incidence data to a standard population age structure. The age-standardized prevalence rate (ASPR) represents the age-standardized number of current cases (including new and past cases) of a disease per 100,000 population, and it is also adjusted on the basis of the standard population age structure. Disability-adjusted life year (DALY) is used to measure the loss of years of healthy life due to disease, disability, or premature death. The year-standardized prevalence rate (YLD) represents nonfatal health loss due to disease or injury, quantifying the decline in quality of life through disability weights (0 = fully healthy, 1 = death), and the year of life lost (YLL) represents life expectancy loss due to premature death, which is calculated as the difference between the target survival age (e.g., 86 years as used in the GBD) and the actual age of death. The health-adjusted life expectancy (HALE) represents the average number of years in which the population is expected to live in a fully healthy state after considering the impact of disability, serving as a core indicator of comprehensive quality of life. DALY rate indicates the total health loss caused by disease, integrating both fatal and disabling burdens (YLL + YLD). The age-standardized disability-adjusted life year rate (ASDR) represents the age-standardized total healthy life years lost per 100,000 people for a specific disease or injury. The annual percentage change (APC) indicates the average annual change rate of disease burden indicators (e.g., IR) within a specific period, reflecting short-term trends. The average annual percentage change (AAPC) represents the overall average change rate of disease burden indicators over the entire study period (e.g., 1990–2021). The uncertainty interval indicates that there is a 95% probability that the true value falls within this range (7).

### 2.2 Data sources

Data on the burden of disease for NP in China and the G20 countries (including all countries that are currently members of the G20, e.g., Brazil, France, Germany, etc., and 19 countries) from 1990 to 2021 were obtained from the GBD 2021 public database (7). The GBD 2021 Burden of Disease and Injury analyses used 100,983 data sources to estimate the number of YLD, YLL, DALY, and HALE for 371 diseases and injuries (7). The GBD 2021 Burden of Disease and Injury analyses also used 100,983 data sources, including HALE (12). Data were extracted from vital registration systems, verbal autopsies, population censuses, household surveys, disease-specific registries, health service linkage data, and other sources and ranged between the 2.5th and 97.5th percentile values to generate 95% UIs for all final estimates (12). The AAPC and APC calculations were analysed by applying the joinpoint regression model to the incidence rate, prevalence rate, and DALY rate for China and the G20 countries. Ethical approval was not needed, as the human subjects were not directly involved.

### 2.3 Joinpoint regression model

The joinpoint regression model was first proposed by Kim et al. (15) in 2000. The model constructs segmented regressions based on the temporal characteristics of the disease distribution and fits and optimizes trends to the data points in each segment, which allows an in-depth study of disease variance characteristics specific to different intervals on a global time scale. The equation for the log-linear model is as follows: $E[y|x]=e^{\beta_{0}+\beta_{1}x+\delta_{1}{\left(x-\tau_{1}\right)}^{+}}+\ldots +\delta_{k}{\left(x-\tau_{k}\right)}^{+}$ , where *y* represents the disease incidence rate, prevalence rate, or DALY rate; *x* represents the year; $\beta_{1}$ represents the regression coefficient; *k* represents the number of connection points; $T_{k}$ represents unknown connection points; $a^{+}=a$ for a > 0 and 0 otherwise. The model results can be summarized via the metrics of APC and AAPC. The APC is given by $\mathrm{APC}=[\frac{y_{x+1}-y_{x}}{y_{x}}]*100\%(e^{\beta_{1}}-1)*100\%$ . The APC is calculated using the formula that evaluates the trend of the independent intervals of the segmented function, and the AAPC is calculated using the formula $\text{AAPC}=(e^{\sum w_{i}\beta_{i}/\sum w_{i}}-1)*100\%$ , which evaluates the average trend over the entire study interval. AAPC and its corresponding 95% confidence interval (CI) were calculated using joinpoint software (version 4.9.1.0, National Cancer Institute, Rockville, MD) to identify trends in disease burden (15).

### 2.4 Autoregressive integral moving average model

The autoregressive integrated moving average model consists of an autoregressive (AR) model and a moving average (MA) model. The basic assumption is that the data series are transient random variables whose autocorrelation can be characterized by the autoregressive integral moving average model (ARIMA) model and that future values can be predicted from past values. The equation is expressed as $Y_{t}=\varphi_{1}Y_{t-1}+\varphi_{2}Y_{t-2}+\ldots +\varphi_{p}Y_{t-p}+e_{t}-\theta_{1}e_{t-1}-\ldots -\theta_{q}e_{t-q},$ where $\varphi_{1}Y_{t-1}+\varphi_{2}Y_{t-2}+\ldots +\varphi_{p}Y_{t-p}+e_{t}$ is the AR model part, $e_{t}-\theta_{1}e_{t-1}-\ldots -\theta_{q}e_{t-q}$ is the MA model part, $Y_{t-p}$ is the observation at cycle ( t−p ), pandq denotes the model order of the AR and MA, and $e_{t}$ is the random error at cycle *t* (16). The time series in the ARIMA model should be a smooth stochastic series with a zero mean. The time series in the ARIMA model should be a smooth stochastic series with a zero mean. At the heart of the ARIMA model is the differencing process, which converts a nonstationary series into a stationary series, allowing for more efficient modelling. To assess the stability of the differenced series, we used autocorrelation (ACF) and partial autocorrelation (PACF) plots. The optimal model was selected based on the Akaike information criterion (AIC) values using the auto.arima() function. This function applies different ARIMA models to univariate time series data and determines the optimal model based on the constraints provided (17).

### 2.5 Statistical analyses

ASIR, ASPR, and ASDR with 95% UI for NPs in China and G20 countries were reported according to age and sex. All rates are reported per 100,000 people. All the statistical tests were two-sided, with *p* < 0.05 indicating statistically significant differences.

Joinpoint regression analyses were used to determine changes in time trends in the NP disease burden. Significant changes in time points were tested using Monte Carlo substitution. The AAPC was calculated for the entire period analysed from 1990 to 2021, and the APC was calculated for each year of model partitioning. The APC and AAPC were used to indicate trends in the ASIR, ASPR, and ASDR for NP in China and the G20 countries. The hypothesis test of whether AAPC or APC is significantly different from zero. APC or AAPC >0 indicates that the period or the whole period has an upwards trend, and APC or AAPC <0 indicates that the period or the whole period has a downwards trend. Joinpoint regression software (version 5.3.0; National Cancer Institute, Division of Statistical Research and Applications) was used.

The ARIMA (*p*, *d*, *q*) model was applied to predict the trend of the NP incidence rate in men and women from 2021 to 2051 in China. The letters p, d, and q represent the order of autoregression, the degree of difference, and the order of the moving average, respectively (18). The ARIMA equation is expressed as follows:


$$\begin{array}{c} Y_{t}=\alpha +\phi_{1}⊗Y_{t-1}+\phi_{2}⊗Y_{t-2}+\ldots +\mathrm{\phi p}⊗Y_{t-p}+\varepsilon_{t}\\ +\theta_{1}⊗\varepsilon_{t-1}+\ldots +\theta_{q}⊗\varepsilon_{t-q} \end{array}$$

where ϕ and θ are autoregressive and moving average parameters, respectively. It denotes the difference time series, and $\varepsilon_{t}$ is the value of the stochastic excursion at time *t*. a is a constant. The construction of the model requires the following steps. First, the augmented Dickey–Fuller (ADF) test is used to determine if the series is smooth. If the results of the ADF test are significant, the series is stable. Second, the parameters of the ARIMA model are approximately determined by the graphs of the autocorrelation function (ACF) and partial autocorrelation function (PACF). The ARIMA function is used to select the best model with the lowest BIC and the highest *R*^2^ (coefficient of determination, a statistic that indicates the goodness of fit of the model). The Ljung–Box *Q* test of residuals, ACF, and PACF is used to determine whether the residuals of the optimal model satisfy the requirements of the white noise series. Finally, after the constructed model was tested for white noise and passed, we applied the model to predict the incidence rate of NP in men and women from 2021 to 2051. Analyses and plots using ARIMA were performed using R 4.4.2 software (R Development Core Team) with the software packages “forecast,” “series,” and “ggplot2.” Statistical significance was considered at *p* < 0.05.

## 3 Results

Table 1 presents the number of cases, incidence rate, prevalence rate, ASIR, ASPR, ASDR, and AAPC from 1990 to 2021 for China and the G20 countries in 1990 and 2021. The data in parentheses are 95% UIs.

**Table 1:** All age cases, age-standardized rates, and average annual percent change (AAPC) of incidence, prevalence, and DALYs for NP in 1990 and 2021: a comparative analysis across G20 and China.

Location	Measure	1990		2021		1990–2021 APPC
		All-ages cases	Age-standardized rate per 100,000	All-ages cases	Age-standardized rate per 100,000	
		*n* (95% UI)	*n* (95% UI)	*n* (95% UI)	*n* (95% UI)	*n* (95% UI)
G20	Incidence	17,987,540 (14,161,240, 22,167,421)	507.46 (400.1, 622.73)	28,711,727 (22,507,743, 35,544,932)	507.19 (398.38, 617.72)	0.004 (−0.0575 to 0.0654)
	Prevalence	83,245,746 (64,671,470, 102,376,153)	2397.31 (1882.44, 2938.47)	137,669,360 (109,047,105, 169,316,233)	2362.64 (1856.22, 2892.6)	−0.0409 (−0.1047 to 0.0229)
	DALYs	8,311,923 (5,540,542, 11,852,981)	238.33 (160.06, 338.55)	13,611,347 (9,077,692, 19,074,179)	234.58 (156.78, 334.4)	−0.0411 (−0.1188 to 0.0365)
China	Incidence	6,192,332 (4,778,922, 7,748,853)	558.53 (441.06, 693.01)	10,292,099 (8,062,751, 13,039,595)	567.23 (448.5, 699.8)	0.0505 (0.0472–0.0539)
	Prevalence	26,530,055 (20,347,966, 32,917,309)	2479.42 (1942.66, 3042.93)	48,377,404 (37,665,091, 60,063,296)	2549.87 (2007.89, 3141.64)	0.0903 (0.0874–0.0933)
	DALYs	2,675,172 (1,740,002, 3,911,199)	248 (163.93, 353.44)	4,807,593 (3,155,340, 6,903,310)	254.77 (166.89, 357.93)	0.0882 (0.0762–0.1002)

### 3.1 Analysis of the number of people affected by the disease in different age groups and sexes in 2021, the prevalence rate, the number of cases, and the incidence rate

**Figure 1:**
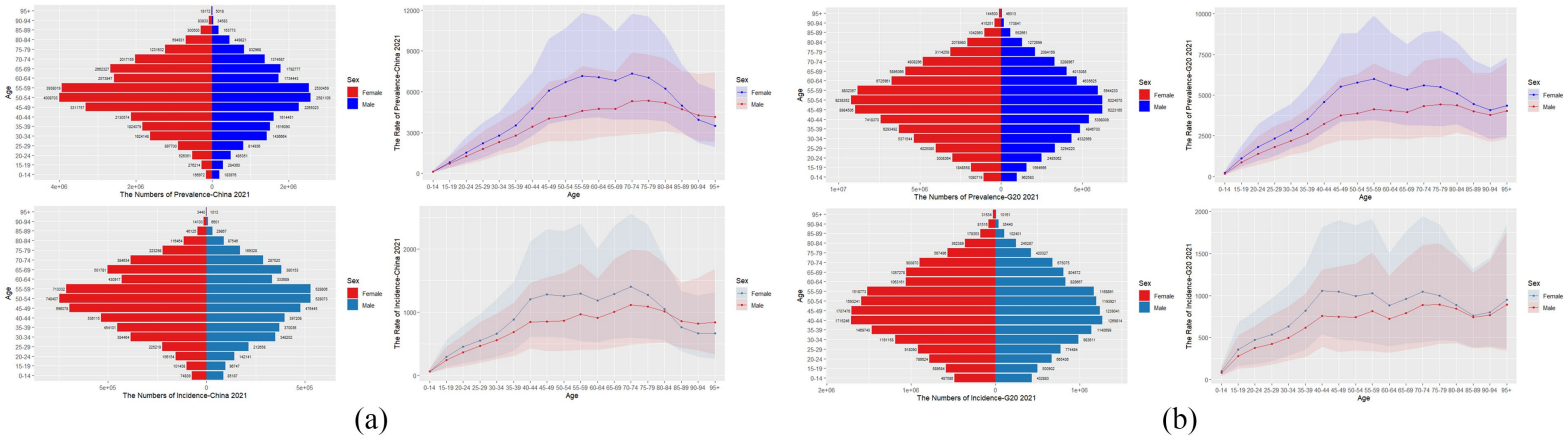
This figure represents the prevalence, prevalence rate, incidence, and incidence rate of NP by age in China **(a)** and G20 countries **(b)** in 2021, with shaded areas indicating 95% UI, including overlap between men and women.

As shown in Figure 1, the incidence and prevalence of NP in China and the G20 countries in 2021 will increase with age, peaking in the 70–74, 40–44, and 55–59 age groups, respectively. In all age groups, the incidence rate and prevalence rate of NP were significantly higher in women than in men, especially in the 45–49 and 50–54 age groups. In addition, the incidence rate and prevalence rate are decreasing in China after the age of 90 but are increasing in the G20 countries. In 2021, 4.48 million (95% UI: 3.48–5.68 million) incident cases and 20.10 million (15.68–25.11 million) prevalent cases of NP were noted in China among men, whereas higher estimates were noted for women, with 5.81 million (4.58–7.34 million) incident cases and 28.27 million (21.87–35.24 million) prevalent cases (see Supplementary Table 1). Similarly, across G20 nations, the NP burden demonstrated a consistent woman predominance. In total, 12.49 million (9.71–15.47 million) incident cases and 57.34 million (44.97–70.12 million) prevalent cases were reported for men, whereas higher figures were noted for women, with 16.22 million (12.84–20.07 million) incident cases and 80.32 million (63.61–99.08 million) prevalent cases. Both in China and in the G20 countries, the incidence and prevalence of NP disease were significantly greater in women than in men. These results suggest that in 2021, the burden of NP disease remained high in both sexes in China and the G20 countries but was more severe in women than in men.

### 3.2 Comparative epidemiology of NP (1990–2021): China vs. G20 countries

**Figure 2:**
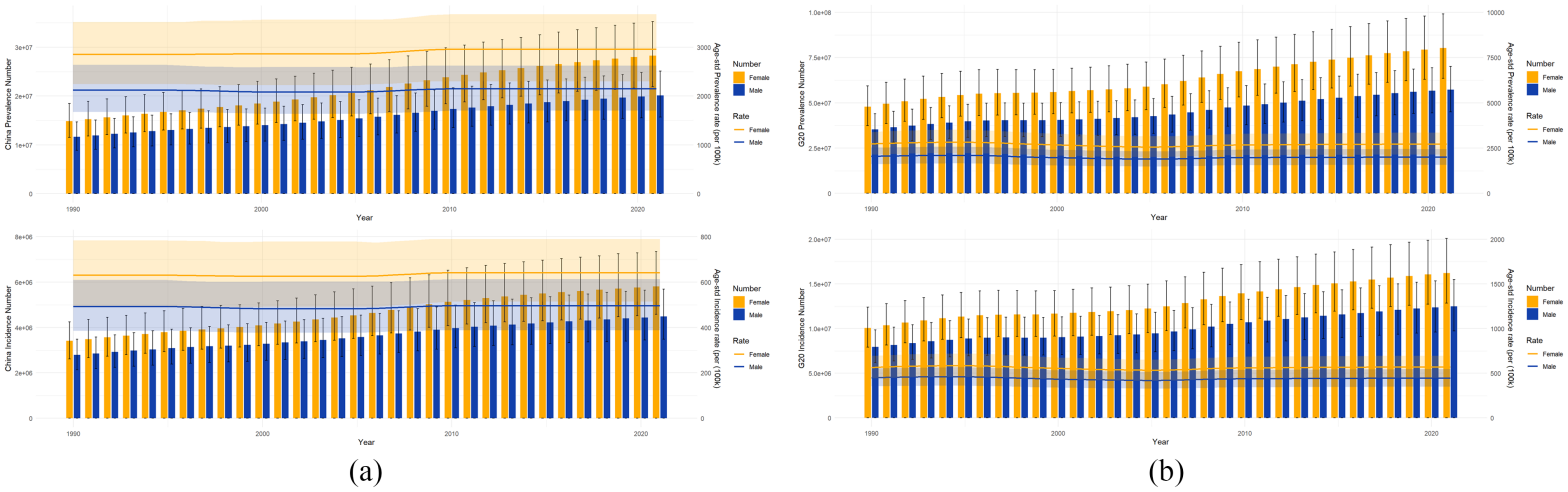
This figure represents the number of prevalent cases, ASPR, number of incidence cases, and ASIR for men and women in China **(a)** and the G20 countries **(b)** from 1990 to 2021. The blue and yellow shaded areas indicate 95% UI, and the grey shaded areas indicate overlap between men and women.

As shown in Figure 2,

1. ASPR

China: ASPR increased significantly from 2,479 (95% UI: 1,942–3,042) per 100,000 population to 2,549 (2,007–3,141) per 100,000 population (*p* < 0.01).

G20 countries: ASPR showed a minor decline from 2,397 (1,882–2,938) per 100,000 population to 2,362 (1,856–2,892) per 100,000 population (*p* > 0.05, nonsignificant).

2. Incident cases & ASIR

China: ASIR increased from 558 (441–693) per 100,000 to 567 (448–699) per 100,000. Annual incident cases: surged 66.1% [6.20 million (4.77–7.74) to 10.30 million (8.06–13.03)].

G20 countries: ASIR remained stable (*p* = 0.12), but absolute incident cases increased 59.7% [17.98 million (14.16–22.16) to 28.71 million (22.50–35.54)].

3. Prevalent cases & sex-specific disparities

China: The number of cases increased from 26.50 million (20.34–32.91) to 48.40 million (37.66–60.06) (+82.6%).

G20 countries: The number of cases increased from 83.20 million (64.67–102.37) to 137.70 million (109.04–169.31) (+65.5%).

### 3.3 Joinpoint regression analysis

**Figure 3:**
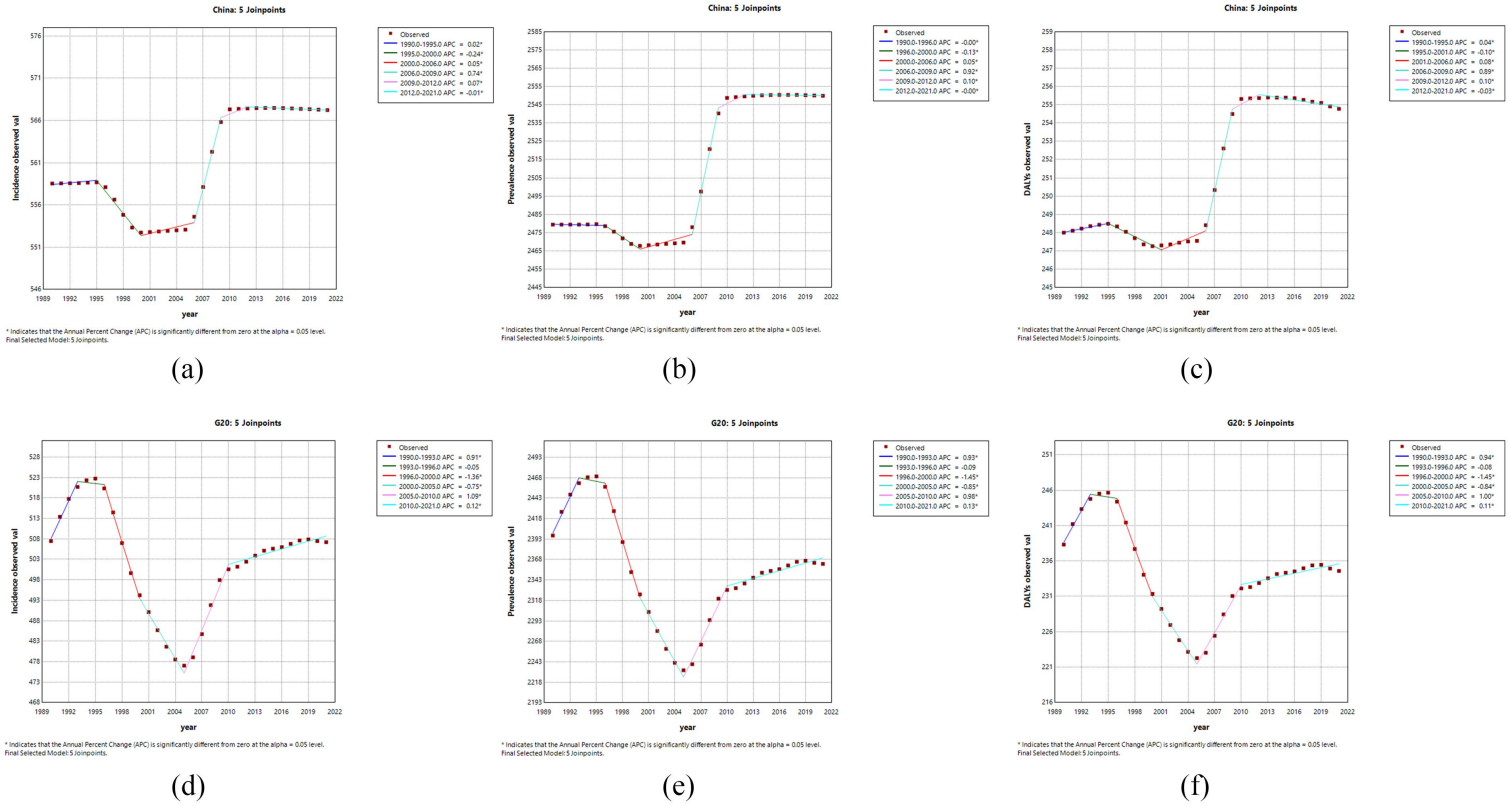
This figure shows the APC of the ASIR, ASPR, and ASDR for NPs in China and G20 countries from 1990 to 2021 (* indicates a *p*-value <0.05, which is significantly different from 0); APC <0 shows a decreasing trend, and APC >0 shows an increasing trend. Plots for ASIR **(a)**, ASPR **(b)**, and ASDR **(c)** for China, and ASIR (**d**), ASPR **(e)** and ASDR **(f)** for the G20 countries are shown.

As shown in Figure 3, the ASIR, ASPR and ASDR of NP in China from 1990 to 2021 all showed an overall increasing trend, with AAPCs of 0.0505% (95% UI = 0.0472–0.0539), 0.0903% (0.0874–0.0933), and 0.0882% (0.0762–0.1002), respectively (Table 1). For NP patients, the APCs of the ASIR, ASPR, and ASDR showed significant upwards trends from 2006 to 2009, with values of 0.74, 0.92, and 0.89%, respectively, which were significantly different from 0. In contrast, the APCs of the ASIR, ASPR, and ASDR showed clear downwards trends from 1995 to 2000, with values of −0.24, −0.13%, and −0.10%, respectively. In addition, from 1990 to 2021, the overall AAPC of the ASIR for the G20 countries showed an increasing trend, at 0.004% (−0.0575–0.0654) (Table 1). Additionally, the APC exhibited a significant upwards trend from 1990 to 1993, at 0.91%, and a significant downwards trend from 1996 to 2000, at −1.36%. The ASPR and ASDR AAPCs both showed an overall downwards trend, with respective values of −0.0409% (−0.1047–0.0229) and −0.0411% (−0.1188–0.0365) (Table 1), and the APCs both showed a significant upwards trend from 2005 to 2010, with respective values of 0.98 and 1.00%. The APCs both showed a significant downwards trend from 1996 to 2000, both at −1.45%, and both values were significantly different from 0.

### 3.4 Projected incidence of neck pain in China (2021–2051)

**Figure 4:**
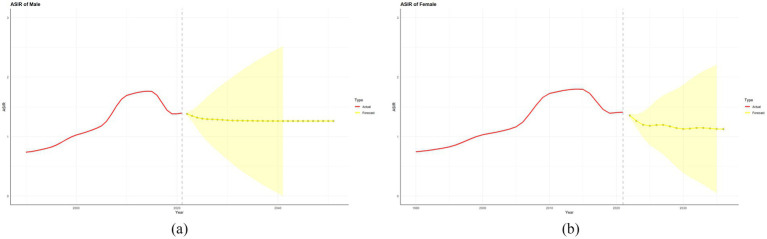
This figure shows the actual and predicted incidence rates of NP for men **(a)** and women **(b)** in China from 1990 to 2021 and up to 2051. Here, the red solid line represents the actual value, the yellow dashed line represents the predicted value, and the yellow shaded area represents the 95% UI.

As shown in Figure 4, the ASIR of NP in China is projected to remain stable at 0.57% (95% UI: 0.49–0.65%) from 2021 to 2051 (*p* = 0.78, time-trend regression). In terms of sex-specific trends, we found that the incidence for women is projected to significantly decline from 0.64% (0.55–0.73%) in 2021 to 0.50% (0.43–0.58%) in 2051 (*p* = 0.01), reflecting a 0.14% reduction (relative decrease: 21.9%). There was no statistically significant change in the rates for men (*p* = 0.92), with the incidence rate predicted to remain stable at 0.50% (0.42–0.58%) (Figure 4).

## 4 Discussion

Over the past 31 years, we have observed significant increases in the incidence rate, prevalence rate, and DALY rate of NP in China. In 2021, the number of NP patients in China reached 48,377,404, and the number of patients in G20 countries reached 137,669,360. This finding reflects the severity of the disease burden of NP in China and even in G20 countries. Compared with the NP burden in the G20 countries, the incidence rate, prevalence rate, and DALY rate of NP in China are significantly higher than those estimated for the G20 countries. In addition, we observed that the ASIR, ASPR, and ASDR AAPCs of both man and woman NP patients increased in China between 1990 and 2021. In contrast, the G20 countries presented increases in the ASIR and ASDR AAPCs but decreases in the G20 ASPR AAPCs. We believe that several reasons explain these phenomena. First, before 1995, the degree of mechanization in China and the world was relatively low, and production was mainly achieved with manual labour and manual operation, especially in some less developed countries and regions; thus, a large number of NP patients performed manual labour (19). However, as the degree of mechanization and automation around the world has increased significantly, the income level and health awareness of people in China and the G20 countries have generally increased, and people are more likely to choose occupations with light labour, which may explain the decline in the APCs for the ASIR, ASPR, and ASDR of NPs in China and the G20 countries after 1995 (5). Second, after 2000, given the rapid development of China’s economy at that time, the number of people with jobs requiring light labour with high incomes increased. These individuals spent most of their time in front of computers in constant postures. Combined with the severe situation of an ageing population (19), these factors may also be the main factors explaining the increasing trends in NP ASIR, ASPR, and ASDR in the APCs. Therefore, we must continue increasing investment in the prevention and treatment of NP.

Moreover, we found that the age-standardized NP ASIRs and ASDRs in China have also increased over the past 31 years. These findings suggest that NP changes and increases in severity at older ages. The odds that psychological risk factors contribute to NP increase with age, e.g., chronic stress, lack of social support, anxiety, and depression are important risk factors for NP (20). Certainly, degenerative cervical instability in older adults is first associated with NP but has not received sufficient attention (21). With the advancement of current medical technology, more NP patients can improve their quality of life by reducing the risk of NP through a combination of Chinese and Western medicine. However, in China, many NP patients do not have access to combined Chinese and Western medicine treatments, especially in areas with more limited medical resources, where NP is not categorized into neuropathic or nonneuropathic pain, and this information needs to be used to guide NP examinations (necessity of imaging) and treatment decisions (5). We found that the incidence rate, prevalence rate, and DALY rate of NP were higher in women than in men in China and the G20 countries. We consider the possibility that women in China and G20 countries may be responsible for more domestic activities or exhibit physiological and structural differences (22). Ostergren et al. (23) noted that the influence of psychosocial factors was more prominent in women, which may be due to the synergistic effect of psychosocial and biological factors leading to an increased risk of shoulder and NP in women. In addition, Hey et al. (24) reported that being a woman was the only identifiable potential risk factor for the prevalence of NP in Singaporeans that may have contributed to the higher incidence rate, prevalence rate, and DALY rate of NP in women.

Monitoring disease prevalence and predicting trends is an important part of disease prevention and control, and the ARIMA model predicts a decreasing trend in the incidence rate of NP in Chinese women over the next 30 years. However, the trend of the NP incidence rate in man subjects was estimated to be insignificant based on the predicted values. This prediction suggests that the risk of NP in women may be declining, possibly due to the current critical situation of an ageing population and the declining birth rate in China (19). Despite clear evidence that NP is a long-term problem characterized by recurrent episodes, little attention has been given to studying effective strategies to prevent NP (25); this is where the large discrepancy between the NP incidence rate, prevalence rate, and DALY rate and low awareness and treatment may partly explain the extent of the increase in the NP disease burden that has continued to occur in recent years. Most studies have investigated interventions for the treatment of NP, and in one review, it was reported that exercise programs also appear to have the potential to prevent NP episodes (26). NP, whether acute or chronic, was also mentioned in another paper as typically being treated through physical or manipulative therapies. These include ultrasound; ice or heat packs; electrical stimulation; avoidance of pain-producing activities; traction; and passive activities. These “conventional” interventions may have some short-term effects in reducing pain and improving neck range of motion; however, there are no long-term effects. On the other hand, exercise has been associated with long-term positive effects (27). This type of literature provides good methods for diagnosing NP and preventing chronic disability (28). There is also another study in the literature that mentions the role of using Eastern and Western manipulation for NP (1). Gonzalez-Alvarez et al. (29) examined lifestyle interventions (e.g., exercise, diet, probiotics, and electroacupuncture) and their effects on the regulation of the gut flora in patients with widespread chronic pain. Another study conducted by Cuenca-Zaldívar et al. (30) explored the effects of climatic variables (e.g., barometric pressure and air temperature) on the frequency of visits for chronic musculoskeletal pain in Spanish primary care. In addition, Pereira et al. (31) reported that workplace interventions combining ergonomics and neck-specific exercises may provide potential long-term benefits for sick leave rates and health-related productivity losses among general office workers, as well as for office workers with NP. Furthermore, a multicentre randomized clinical trial of Italian surgeons demonstrated the effectiveness of a global program based on ergonomic applications in the operating room and specific lower back exercises (32). In conclusion, a better understanding of the factors influencing NP and effective strategies to prevent NP could provide public health workers with a focus on NP research.

This study has several advantages. To the best of our knowledge, this study investigated temporal trends in the NP incidence rate, incidence rate, and DALY rate by sex in China and G20 countries. Second, GBD 2021 uses a uniform and standardized methodology in its data analysis techniques, making these estimates comparable across time. Moreover, not only the change over the entire period (as assessed by the AAPC) but also that over each segmented period (as assessed by the APC) is determined using a joinpoint regression model. Finally, the use of the ARIMA model to predict the incidence rate of NP in China over the next 30 years is important for further prevention of NP. Moreover, Fernández-Carnero et al. (33) explored the predictors of patient satisfaction following the use of Mulligan activity therapy for chronic NP, demonstrating how clinical predictors can be statistically modelled to improve treatment accuracy and outcome prediction in musculoskeletal pain syndrome patients, and the combination of these two factors is also important for furthering the prevention of NP in the future.

However, this study has several limitations. First, the data source in this study was GBD 2021; these values were obtained mainly from modelling data using the process in DisMod-MR 2.1 rather than direct measurements, leading to unavoidable bias, as previously described (7). However, in GBD 2021, several adjustments were used to minimize bias, and the reliability of this source has been confirmed by the previous literature and IHME annual reports. In addition, the interpretation of the results focuses on the population level rather than the individual level, which may lead to ecological fallacies. For example, certain occupations, such as office and computer workers, manual labourers, and health care workers, have been found to have a high incidence rate of NP in some studies, but the main workplace factors associated with this condition are low job satisfaction and a perceived poor workplace environment (34). There is also uncertainty in predicting the future incidence rate of NP in China. In addition, with the further development of artificial intelligence, people, such as working for long hours in front of computers and maintaining static postures for long periods, may lead to the development of NP (35). A recent study on the burden of NP in the general population of China revealed that NP is a serious public health problem in the general population of China, especially in the central and western regions, with an overall increasing trend over the past three decades (36), which is generally consistent with the results of the present study and reinforces the top priority of the disease burden of NP in China. Subsequent studies predicting the future disease burden of NP in China can be further examined and confirmed when the GBD database is updated.

Therefore, the results of this study can only provide policy-makers with a general direction to address the disease burden of NP. To develop policies specific to a particular country or region, more targeted fieldwork and survey-based research are required. A recent Lancet report emphasized the need for legislation to incorporate a balance between local needs and global frameworks (37).

## 5 Conclusion

In conclusion, NP is a serious public health challenge for people in China and G20 countries. In the past, the ASIR, ASPR, and ASDR increased. Given the current serious situation of population ageing and the demographic indices of China and the top G20 countries in the world, the burden of NP may be even more serious in the future. Therefore, improving the precise clinical diagnosis and targeted treatment of NP has the potential to not only reduce the unnecessary waste of medical resources but also reduce the recurrence of NP. Therefore, to reduce the NP disease burden in the future, policy-makers can formulate policies that consider factors such as the different ages and occupations of local individuals and the climate of the local environment. In particular, some high-risk occupations and harsh climatic environments should be given more legislative protection, such as personalizing the implementation of ergonomic exercises, improving the working environment and reducing working hours as much as possible, to address the root causes of the burden of NP on people. The burden brought by NP to the people.

## Data Availability

The original contributions presented in the study are included in the article/Supplementary material, further inquiries can be directed to the corresponding author.
